# Characterization and distribution of viridans group streptococci isolated from infectious endophthalmitis and keratitis

**DOI:** 10.5935/0004-2749.20200087

**Published:** 2024-02-11

**Authors:** Katiane Santin, Paulo José Martins Bispo, Talita Trevizani Rocchetti, Lucas Denadai, Willames Marcos Brasileiro da S. Martins, Mirian Silva do Carmo, Ana Luisa Hofling-Lima

**Affiliations:** 1 Ophthalmology and Visual Sciences Department, Universidade Federal de São Paulo, São Paulo, SP, Brazil; 2 Laboratório Especial de Microbiologia Clínica, Universidade Federal de São Paulo, São Paulo, SP, Brazil; 3 Laboratório Alerta, Division of Infectious Diseases, Department of Internal Medicine, Universidade Federal de São Paulo, São Paulo, SP, Brazil

**Keywords:** Endophthalmitis, Keratitis, Eye infections, bacterial, Streptococcal infections, Viridans streptococci/isolation & purification, Drug resistance, microbial, Fluoroquinolones, Endoftalmite, Ceratite, Infecções oculares bacterianas, Infecções estreptocócicas, Estreptococos viridans/ isolamento & purificação, Resistência antimicrobiana a medicamentos, Fluoroquinolonas

## Abstract

**Purpose:**

The aims of this study were to characterize alpha-hemolytic streptococci among isolates from cases of infectious endophthalmitis and keratitis and to determine their distributions.

**Methods:**

The sample included 27 and 35 nonduplicated isolates of alpha-hemolytic streptococci recovered from patients with infectious endophthalmitis (2002-2013) and keratitis (2008-2013), respectively. Isolates were identified by the optochin susceptibility and bile solubility tests, using a biochemical identification system. The minimum inhibitory concentration was determined by the broth microdilution method. Molecular identification was performed by analyses of three constitutive genes and the complementary multilocus sequence. The molecular epidemiology of *Streptococcus pneumoniae* was investigated using multilocus sequence typing, and the presence of the capsular polysaccharide-encoding gene was assessed using conventional polymerase chain reaction. Outcomes were evaluated using the patients’ medical records.

**Results:**

Phenotypic tests differentiated *S. pneumoniae* from other alpha-hemolytic streptococci, consistent with later molecular identifications. *Streptococcus oralis* was significantly prevalent among the endophthalmitis isolates, as was *S. pneumoniae* in the keratitis isolates. High levels of susceptibility to antibiotics were observed, including vancomycin, cephalosporins, and fluoroquinolones. High genetic variability was detected among the 19 *S. pneumoniae* strains, with 15 predicted to be encapsulated. The medical records of patients with infectious endophthalmitis were reviewed (n=15/27; 56%), and final visual acuity was assessed in 12 cases (44%). Many patients progressed to a final visual acuity state of “no light perception” (6/12; 50%), “light perception” (3/12; 25%), or “hand motion” (1/12; 8%). The medical records of patients with infectious keratitis were also reviewed (n=24/35; 69%), and final visual acuity was assessed in 18 cases (51%). Similarly, most patients progressed to a final visual acuity state of “no light perception” (6/18; 33%), “light perception” (1/18; 6%), or “hand motion” (6/18; 33%). Overall, the majority of patients progressed to a final visual acuity state of “no light perception” (12/30), “light perception” (4/30), or “hand motion” (7/30).

**Conclusions:**

The distribution of alpha-hemolytic streptococci in ocular infections suggested the presence of a species-specific tissue tropism. The prognoses of patients with ocular streptococcal infections were highly unfavorable, and antibiotic resistance did not contribute to the unfavorable clinical progressions and poor outcomes.

## Abstract (pt)

**Objetivo:**

O objetivo deste estudo foi caracterizar os estreptococos alfa-hemolíticos isolados de endoftalmite infecciosa e ceratite e determinar sua distribuição.

**Métodos:**

A amostra incluiu 27 e 35 isolados não-duplicados de estreptococos alfa-hemolíticos recuperados de pacientes com endoftalmite infecciosa (2002-2013) e ceratite (2008-2013), respectivamente. Os isolados foram identificados pelos testes de suscetibilidade à optoquina e bile solubilidade, utilizando um sistema de identificação bioquímica. A concentração inibitória mínima foi determinada pelo método de microdiluição em caldo. A identificação molecular foi realizada pela análise de três genes constitutivos e análise complementar de sequências multilocus. A epidemiologia molecular do *Streptococcus pneumoniae* foi investigada por tipagem de sequência multilocus, e a presença do gene codificador do polissacarídeo capsular foi avaliada por reação em cadeia da polymerase convencional. Os resultados foram avaliados utilizando os prontuários médicos dos pacientes.

**Resultados:**

Os testes fenotípicos diferenciaram *S. pneumoniae* dos outros estreptococos alpha-hemolíticos, consistentes com identificações moleculares posteriores. *S. oralis* foi significativamente prevalente entre os isolados de endoftalmite, assim como *S. pneumoniae* nos isolados de ceratite. Foram observados altos níveis de suscetibilidade a antibióticos, incluindo vancomicina, cefalosporinas e fluoroquinolonas. Alta variabilidade genética foi detectada entre as 19 cepas de *S. pneumoniae*, com 15 previstas para serem encapsuladas. Os prontuários médicos dos pacientes com endoftalmite infecciosa foram revisados (n=15/27; 56%), e a acuidade visual final foi avaliada em 12 casos (44%). Muitos pacientes evoluiram para um estado final de acuidade visual de “sem percepção luminosa” (6/12; 50%), “percepção luminosa” (3/12; 25%) ou “movimentos de mãos” (1/12; 8%). Também foram revisados os prontuários médicos dos pacientes com ceratite infecciosa (n=24/35; 69%), e a acuidade visual final foi avaliada em 18 casos (51%). Da mesma foram, a maioria dos pacientes evoluiu para um estado final de acuidade visual de “sem percepção luminosa” (6/18; 33%), “percepção luminosa” (1/18; 6%) ou “movimentos de mãos” (6/18; 33%). No geral, a maioria dos pacientes evoluiu para um estado final de acuidade visual de “sem percepção luminosa” (12/30), “percepção luminosa” (4/30) ou “movimentos de mãos” (7/30).

**Conclusões:**

A distribuição de estreptococos alfa-hemolíticos nas infecções oculares sugeriu a presença de um tropismo de tecido específico da espécie. Os prognósticos dos pacientes com infeções oculares por estreptococos foram altamente desfavoráveis e a resistência a antibióticos contribuiu não para as progressões clínicas desfavoráveis e os maus resultados.

## INTRODUCTION

Alpha-hemolytic streptococci comprise a large group of primarily commensal organisms usually found in the mucosae of humans. These streptococci can cause serious infections, such as endocarditis, meningitis, pneu monia, abscesses, and septicemia^(1)^. These organisms have also emerged as important causes of serious and sight-threatening eye infections, such as infectious keratitis and endophthalmitis, especially following intravitreal injections^(2^-^4)^. Streptococcal endophthalmitis and keratitis are usually acute, rapid-onset infections that are aggressive and frequently lead to worse outcomes in comparison with other causative bacteria^(5^-^7)^. These ocular bacterial infections are associated with risk factors such as contact lenses, trauma, surgery, age, dry eye state, chronic nasolacrimal duct obstruction, and previous ocular infection^(8^,^9)^.

In developing countries, most ocular infections are caused by members of the genus *Staphylococcus.* However, members of the genus *Streptococcus* have also been involved in ocular infections, resulting in worse outcomes than staphylococcal infections, especially for post-cataract endophthalmitis^(10)^. At our university, bacterial keratitis is caused by members of the genus *Staphylococcus* (51.7%) (coagulase-negative *Staphylococcus* followed by *Staphylococcus aureus*), *Corynebacterium* spp. (14.1%), *Streptococcus* spp. (9.9%), *Pseudomonas* spp. (6.3%), *Moraxela* spp. (5.5%), *Serratia* spp. (4.2%), *Enterobacter* spp. (1.9%), and others^(11)^. Coagulase-negative staphylococci were the primary agents responsible for bacterial endophthalmitis (56.5%) in our setting, but alpha-hemolytic streptococci were ranked second (15.2%)^(12)^. These organisms are routinely separated into viridans group streptococci (VGS) and *Streptococcus pneumoniae* on the basis of their susceptibility to optochin and bile solubility.

Alpha-hemolytic streptococci comprise several species that are clustered into six major groups: *mutans, salivarius, mitis, anginosus, sanguinis,* and *bovis*^(13)^. *S. pneumoniae* is phylogenetically placed in the *mitis* group^(14)^. Phenotypic identification at the *Streptococcus* species-level is challenging and frequently erroneous^(15)^. The use of only one genetic target for species differentiation is often not reliable, as interspecies recombination events are common in this genus^(16^-^18)^.

The present study aimed to determine the species of alpha-hemolytic streptococci causing endophthalmitis and keratitis and their prevalence patterns, along with their antimicrobial resistance profiles, using a combination of phenotypic and genotypic approaches. In addition, we identified potential factors associated with disease pathogenesis and clinical outcomes.

## METHODS

### Bacterial isolates

We included 62 nonduplicated VGS isolates recovered from patients with endophthalmitis (n=27; 2002-2013) and keratitis (n=35; 2009-2013) seen at the Department of Ophthalmology and Visual Sciences, Federal University of São Paulo, Brazil. After confirmation in the clinical laboratory, when *in vitro* isolates derived from clinical samples showed substantial and pure growth in different culture media and were identified as part of routine laboratory protocols, the specimens were stored at -80°C in tryptic soy broth (TSB) with 15% glycerol. These isolates were recovered for re search purposes from a freezer storage bank for isolates by culturing using commercial 5% sheep blood agar or chocolate agar (PROBAC, São Paulo, Brazil) at 37°C in a 5% CO_2_ atmosphere before testing.

### Patient data

The Federal University of São Paulo Institutional Review Board approved review of the medical records of the patients from whom the isolates were collected. Patient demographics included age and sex, risk factors associated with infection (e.g., surgery, intravitreal injection, and trauma), antibiotic treatment, and outcomes.

### Phenotypic identification

Isolates were initially classified on the basis of biochemical tests regularly used to differentiate *S. pneumoniae* from other alpha-hemolytic streptococci, such as the optochin susceptibility test and bile solubility test using sodium deoxycholate. All tests were performed twice. The control *S. pneumoniae* ATCC49619 strain was used as a reference strain in all tests.

### Antimicrobial susceptibility testing

Minimum inhibitory concentrations were determined by the broth microdilution method using a commercial panel of antibiotics (STP6F, Sensititre Trek; Thermo Scientific, Waltham, MA, USA). Quality control was performed by testing with the *S. pneumoniae* ATCC49619 strain. Interpretive criteria published by the Clinical and Laboratory Standards Institute, document M100-S27, were followed.

### DNA extraction

DNA was extracted using Chelex 100 Molecular Biology Resin (BioRad, Hercules, CA, USA), as previously reported^(19)^. Briefly, fresh isolates grown on 5% sheep blood agar (PROBAC) were cultured in 5 mL of TSB without shaking overnight at 37°C in a 5% CO_2_ atmosphere. An aliquot of 1 mL was centrifuged for 5 min at 13,000 rpm (5415R; Eppendorf, Hamburg, Germany), and the cells were washed twice using 1× phosphatebuffered saline. The pellets were resuspended in 300 µL of 10% Chelex 100 resin and incubated for 30 min at 95°C. After centrifugation for 5 min at 13,000 rpm, a 1 µL aliquot of the supernatant was collected, diluted 1:10 (v/v), and used for polymerase chain reaction (PCR) amplification.

### Molecular identification

Molecular identification was initially performed by sequencing three constitutive genes, *rpo*B*, sod*A, and *tuf*, as previously reported^(20)^. The PCR products were purified (QIAquick PCR Purification Kit; Qiagen, San Diego, CA, USA), and both strands were sequenced using the BigDye fluorescent terminator with an ABI 3000 genetic analyzer (Applied Biosystems, Foster City, CA, USA). The sequences obtained were edited using SeqMan (DNASTAR, Madison, WI, USA). Sequence identities were searched for in GenBank using the BLAST tool (https://blast.ncbi. nlm.nih.gov). Isolates without identification agreement for these three genes were subjected to multilocus sequence analysis (MLSA), as previously described^(20)^. A neighbor-joining tree was constructed on the concatenated MLSA alleles using MEGA software, version 5.0 (https://mega.software.informer.com/5.0/).

### Multilocus sequence typing (MLST) scheme for *Streptococcus pneumoniae*

All *S. pneumoniae* isolates (n=19) were subjected to MLST, as previously described^(21)^. The MLST procedure and details of the full analysis are available at the *S. pneumoniae* MLST website (https://pubmlst.org/spneumoniae/). Sequence types (STs) were assigned using the same database, and clonal complexes (CCs) were determined using the goeBURST algorithm (http://www.phyloviz.net/goeburst/).

### Detection of the capsular polysaccharide (*cps*)-encoding gene

PCR amplification of an internal product of the *cps*A gene (654 bp) was performed using the following primer pairs: forward, 5′-TACTAGTTGCCTTGGTAGG-3′; reverse, 5′-CGATTGGTACATAGGCATCA-3′. A 25 µL reaction mix was prepared using 12.5 µL of 2× GoTaq Green Master Mix (2.5 U GoTaq DNA Polymerase, 400 µM of each dNTP, 3.0 mM MgCl_2_, and reaction buffer) (Promega, Madison, WI, USA), 0.5 µL of each primer, 1 µL of DNA template, and sterile Milli-Q water. The PCR conditions were as follows: initial denaturation at 95°C for 10 min; 30 cycles of 95°C for 60 s, 51°C for 60 s, and 72°C for 60 s; and a final extension at 72°C for 10 min. The amplicons were separated using agarose gel electrophoresis.

### Statistical analyses

Fisher’s exact test was used to analyze the distributions of the infectious species that caused endophthalmitis and keratitis. Simpson’s diversity index (SDI) was used to evaluate *S. pneumoniae* diversity.

## RESULTS

### *Streptococcus oralis* and *Streptococcus pneumoniae* are the leading causes of ocular streptococcal infections

To determine the most common species of alpha-he molytic streptococci causing ocular infections in our setting, we used a combination of phenotypic and genotypic tests to identify 62 alpha-hemolytic streptococcal isolates from patients with endophthalmitis or keratitis. On the basis of the optochin susceptibility and bile solubility tests, 30.6% (n=19) of the isolates in our collection were identified as *S. pneumoniae*, and the other 69.4% (n=43) were placed in the viridans group.

**Figure 1:**
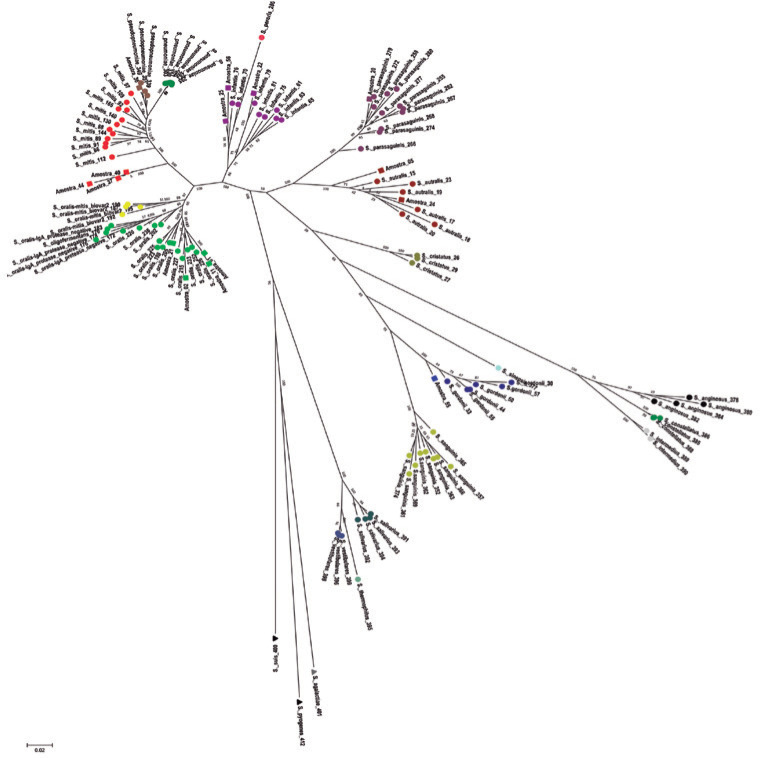
Phylogenetic tree displaying the clonal relationship between the strains under identification in this study (squares) and reference strains (circles) stored in the eMLSA database (http://viridans.emlsa.net/). The phylogenetic tree was constructed using the neighbor-joining algorithm and concatenated sequences of seven housekeeping gene fragments (*map, pfl, ppaC, pyk, rpoB, sodA*, and *tuf*). The outgroup was represented by *Streptococcus agalactiae, S. pyogenes*, and *S. suis* (triangles).

Species-level identifications of the 43 alpha-hemolytic streptococcal isolates were initially performed by analyzing the *rpo*B*, soda,* and *tuf* sequences. Further analysis using a full MLSA scheme^(20)^ was then performed on 15 isolates that were not successfully identified by the initial approach (Figure 1). Phenotypic identification of isolates as *S. pneumoniae* was confirmed molecularly. In total, nine different species were identified in our collection (Table 1). Overall, *S. oralis* (32.2%) and *S. pne umoniae* (30.6%) predominated. The distribution of these species was not random across different diseases. *S. oralis* was significantly prevalent in endophthalmitis (13/27; 48.1%; p=0.0013), whereas *S. pneumoniae* was the leading cause of keratitis (17/35; 48.6%; p=0.0013).

**Table 1:** Distribution of streptococcal species according to molecular identification

Organism	Endophthalmitis (n=27)	Keratitis (n=35)	Total (n=62)
*Streptococcus oralis*	13 (48.1%)	7 (20.0%)	20 (32.3%)
*Streptococcus pneumoniae*	2 (7.4%)	17 (48.6%)	19 (30.6%)
*Streptococcus sanguinis*	6 (22.2%)	1 (2.9%)	7 (11.3%)
*Streptococcus mitis*	0 (0.0%)	5 (14.3%)	5 (8.1%)
*Streptococcus gordonii*	1 (3.7%)	2 (5.7%)	3 (4.8%)
*Streptococcus infantis*	2 (7.4%)	1 (2.9%)	3 (4.8%)
*Streptococcus parasanguinis*	1 (3.7%)	1 (2.9%)	2 (3.2%)
*Streptococcus australis*	2 (7.4%)	0 (0.0%)	2 (3.2%)
*Streptococcus pseudopneumoniae*	0 (0.0%)	1 (2.9%)	1 (1.6%)

### Resistance rates of clinically relevant antibiotics

In general, VGS and *S. pneumoniae* were highly susceptible to commonly used antibiotics (Table 2). Only a small percentage of VGS isolates were resistant to the fluoroquinolones frequently used in ophthalmology, namely, levofloxacin (7.0%) and moxifloxacin (2.3%), whereas all *S. pneumoniae* isolates were susceptible to these drugs. All isolates were susceptible to vancomycin (MIC_90_ ≤0.5 µg/mL), linezolid (MIC_90_ 2 µg/mL), and daptomycin (MIC_90_ 1 µg/mL for VGS and 0.12 µg/mL for *S. pneumoniae*). Using oral penicillin V interpretive breakpoints, the rates of penicillin resistance were 21.1% for *S. pneumoniae* and 16.3% for VGS. However, these isolates were susceptible to other beta-lactam agents tested, including amoxicillin clavulanate and the thirdand fourth-generation cephalosporins ceftriaxone and cefepime. Only 2.3% of VGS isolates were not susceptible to ertapenem and meropenem. The highest rates of resistance among VGS were seen with the macrolides azithromycin (60.5%, MIC_90_ >2 µg/mL) and erythromycin (62.8%, MIC_90_ >2 µg/mL). By contrast, *S. pneumoniae* isolates were sensitive to both drugs. Resistance to trimethoprim-sulfamethoxazole (57.9%) and chloramphenicol (5.3%) was more frequently observed in *S. pneumoniae* than in VGS (39.5% and 0.0%, respectively), whereas resistance to tetracycline (25.6%) and clindamycin (16.3%) was more frequently observed in VGS than in *S. pneumoniae* (10.5% and 0.0%, respectively).

**Table 2:** Antimicrobial susceptibility profile of alpha-hemolytic streptococci isolated from endophthalmitis and keratitis

Antimicrobial agent	VGS (n=43)				*Streptococcus pneumoniae* (n=19)			
	**MIC_50_**	**MIC_90_**	**%S**	**%NS**	**MIC_50_**	**MIC_90_**	%S^*^	%NS^*^
Penicillin^**^	0.06	0.025	83.7	16.3	≤0.03	0.12	78.9	21.1
Amoxicillin clavulanate	≤2/1	≤2/1	100.0	0.0	≤2/1	≤2/1	100.0	0.0
Ceftriaxone	≤0.12	0.25	100.0	0.0	≤0.12	≤0.12	100.0	0.0
Cefepime	≤0.5	≤0.5	100.0	0.0	≤0.5	≤0.5	100.0	0.0
Ertapenem	≤0.5	≤0.5	97.7	2.3	≤0.5	≤0.5	100.0	0.0
Meropenem	≤0.25	≤0.25	97.7	2.3	≤0.25	≤0.25	100.0	0.0
Azithromycin	1	>2	39.5	60.5	≤0.25	≤0.25	100.0	0.0
Erythromycin	2	>2	37.2	62.8	≤0.25	≤0.25	100.0	0.0
Clindamycin	≤0.12	>1	83.7	16.3	≤0.12	≤0.12	100.0	0.0
Chloramphenicol	2	4	100.0	0.0	2	4	94.7	5.3
Trimethoprim-sulfamethoxazole ^*^	1.0/19	4.0/76	60.5	39.5	1.0/19	>4.0/76	42.1	57.9
Tetracycline	≤1	≤1	74.4	25.6	≤1	≤1	89.5	10.5
Levofloxacin	1	2	93.0	7.0	1	2	100.0	0.0
Moxifloxacin^*^	≤1	≤1	97.7	2.3	≤1	≤1	100.0	0.0
Linezolid	1	2	100.0	0.0	1	2	100.0	0.0
Vancomycin	≤0.5	≤0.5	100.0	0.0	≤0.5	≤0.5	100.0	0.0
Daptomycin	0.5	1	100.0	0.0	0.12	0.12	^**^	^**^

NS= nonsusceptible (intermediate or resistant); S= susceptible; VGS= viridans group streptococci.

^*^ *S. pneumoniae* breakpoints were applied for categorization.

** Breakpoints for oral penicillin V were applied.

### The population structure of *Streptococcus pneumoniae* was highly diverse

A deviant and unique clade of unencapsulated *S. pneumoniae* has been identified as the predominant cause of outbreak-related and outbreak-nonrelated conjunctivitis in the United States^(22)^. To determine whether these conjunctivitis strains were present in our setting, we detected *cps*A using a combination of MLST and PCR. We found that most of the *S. pneumoniae* isolates (15/19; 78.9%) tested positive for the *cps*A gene, and these were predicted to be encapsulated strains (Table 3). MLST analysis demonstrated that our collection was highly diverse. In total, 13 different STs were found in our population (SDI=0.982). All STs belonged to different CCs, except for ST66 and ST73, which belonged to CC66. Three isolates (15.8%) were found to have new STs (ST13545, ST13546, and ST13547). Among the isolates lacking the *cps*A gene (n=4), only one (ST2315) was found to be part of the previously reported epidemic conjunctivitis cluster of unencapsulated strains. The other *cps*A-negative isolates belonged to ST1262 (two isolates) and ST11374 (one isolate), which were not reported as part of the conjunctivitis cluster.

**Table 3:** Molecular typing of *Streptococcus pneumoniae* recovered from infectious endophthalmitis and keratitis correlated with the capsular polysaccharide-encoding gene (*cps*A)

Sample	Disease	*cps*A	Sequence type	Clonal complex
26	Endophthalmitis	Negative	2315	2315
27	Endophthalmitis	Positive	6403	698
29	Keratitis	Positive	338	156
32	Keratitis	Positive	13545	2185
43	Keratitis	Positive	42	439
44	Keratitis	Positive	2014	3518
45	Keratitis	Positive	73	66
46	Keratitis	Negative	11374	1106
47	Keratitis	Positive	727	Singleton
48	Keratitis	Positive	72	72
49	Keratitis	Positive	13546	Singleton
50	Keratitis	Negative	1262	1262
55	Keratitis	Negative	1262	1262
56	Keratitis	Positive	66	66
57	Keratitis	Positive	62	62
58	Keratitis	Positive	770	770
59	Keratitis	Positive	62	62
60	Keratitis	Positive	13547	3669
62	Keratitis	Positive	727	Singleton

### Endophthalmitis and keratitis caused by alpha-hemolytic streptococci resulted in poor outcomes

The clinical features, antibiotic treatments, and visual outcomes of patients with endophthalmitis and ker atitis are summarized in tables 4 and 5. Most of the endophthalmitis cases were postoperative following phacoemulsification (n=13; 48.1%). The others were post-traumatic (n=3; 11.1%), post-intravitreal injection (n=2; 7.4%), and endogenous (n=1; 3.7%) or due to corneal perforation (n=1; 3.7%), among other causes. The average age was 53 years (range, 1-89 years). Most of the patients were female (16/27, 59.2%). Most of the patients had vitreous humor collected for culture by means of vitrectomy (n=13) or vitreous tap (n=8), and aqueous humor culture was performed on a smaller number of patients (n=6). In a subset of patients for whom treatment information was available (n=15), the majority (n=14) were treated with intravitreal injections of antibiotics, mainly vancomycin and ceftazidime, as well as topical fluoroquinolones (n=12) and a variety of oral or intravenous antibiotics. Vitrectomy was performed in 10 patients and anterior chamber washing in 2 patients. Corticoid use was reported in 11 cases. Despite prompt clinical and surgical treatment, the patients had poor visual outcomes. Among 12 patients with recorded final visual acuity (VA) outcomes, 1 patient had a final VA of 20/30 and another had a VA of 20/150. The others had final VA scores of “hand motion” (n=1), “light perception” (n=3), and “no light perception” (n=6).

**Table 4:** Demographics, treatment, and clinical outcomes of infectious endophthalmitis cases seen from 2002 to 2013 (n=27)

Characteristic	Result
Age	
Mean (yr)	53
Missing	4(15%)
Gender	
Female	16 (59%)
Male	11 (41%)
Eye	
OD	15(56%)
OS	6 (22%)
Missing	6 (22%)
Sample	
Vitreous humor vitrectomy	13 (48%)
Vitreous humor puncture	8 (30%)
Aqueous humor	6 (22%)
Category	
Postoperative (PHACO)	13 (48%)
Trauma	3 (11%)
Other surgery	2 (7%)
1V1	2 (7%)
Miscellaneous	1 (4%)
Endogenous	1 (4%)
After keratitis	1 (4%)
Unknown	4(15%)
Treatment	
WPP + 1V1 VAN + CTZ; topical MOX	4(15%)
1V1 VAN + CTZ	2 (7%)
AC washing + 1V1 VAN + CTZ; topical MOX and endovenous CEF	1 (4%)
AC washing + IV VAN + AMI; CEF, VAN + MERO; topical MOX	1 (4%)
AC washing + WPP, 1V1 VAN + CTZ; topical MOX; oral CRO + CLIN	1 (4%)
WPP + 1V1 VAN + CTZ; topical GAT	1 (4%)
WPP + 1V1 VAN + CTZ; topical MOX; oral CRO	1 (4%)
WPP + 1V1 and subconjunctival VAN + CTZ; endovenous CEF + GEN; topical MOX	1 (4%)
WPP + IO VAN	1 (4%)
WPP + STX, CEF + MOX	1 (4%)
Topical GAT	1 (4%)
Missing	12 (44%)
Corticoid use	
Prednisone, prednisolone, or dexamethasone	11 (41%)
Missing	16 (59%)
VA outcome	
20/30	1 (4%)
20/150	1 (4%)
HM	1 (4%)
LP	3 (11%)
NLP	6 (22%)
Missing	15(56%)

AC= anterior chamber; HM= hand motion; IO= intraocular; IVI= intravitreal injection; LP= light perception; NLP= no light perception; OD= right eye; OS= left eye; PHACO= phacoemulsification; VA= visual acuity; VVPP= vitrectomy via pars plana. Antibiotics: AMI= amikacin; CEF= cefalotine; CLIN= clindamycin; CRO= ceftriaxone; CTZ= ceftazidime; GAT= gatifloxacin; GEN= gentamicin; MERO= meropenem; MOX, moxifloxacin; STX= sulfamethoxazole-trimethoprim; VAN= vancomycin.

**Table 5:** Demographics, treatment, and clinical outcomes of infectious keratitis cases seen from 2009 to 2013 (n=35)

Characteristic	Result
Age	
Mean (yr)	54
Gender	
Female	22 (63%)
Male	13 (37%)
Eye	
OD	14 (40%)
OS	14 (40%)
OD/OS	1 (3%)
Missing	6(17%)
Predisposing factors	
Corneal transplant	5 (14%)
Soft contact lens wearer	3 (9%)
Bullous keratopathy	3 (9%)
Rigid contact lens wearer	1 (3%)
Allergic conjunctivitis	1 (3%)
Corneal trauma	1 (3%)
Entropium, trichiasis	1 (3%)
Trachoma	1 (3%)
Dry eye syndrome	1 (3%)
Neurotrophic ulcer	1 (3%)
Missing	10 (29%)
Previous surgery	
Phacoemulsification	10 (29%)
Glaucoma	3 (9%)
Retinal detachment	2 (6%)
Treatment	
Topical MOX	6(17%)
MOX, DOX, therapeutic lens	2 (6%)
MOX, DOX	1 (3%)
MOX, therapeutic lens, and AC washing	1 (3%)
MOX, glue, and therapeutic lens	1 (3%)
MOX and ERY ointment	1 (3%)
Topical MOX and GEN, endovenous AMI and CEF	1 (3%)
Topical MOX; CEF and TOB (fortified)	1 (3%)
MOX; subconjunctival injection VAN; oral C1P and cefazoline	1 (3%)
MOX, OFLOX	1 (3%)
OFLOX and therapeutic lens	1 (3%)
OFLOX	1 (3%)
C1P, propamidine, and chlorhexidine	1 (3%)
C1P	1 (3%)
GAT	1 (3%)
CTX and endovenous CLIN	1 (3%)
Missing	13 (37%)
VA outcome	
20/70	1 (3%)
20/100	1 (3%)
20/200	3 (9%)
HM	6(17%)
LP	1 (3%)
NLP	6(17%)
Missing	17 (49%)

The average age of patients with keratitis was 54 years (range, 7-93 years) for 22 females and 13 males. Ophthalmological procedures prior to keratitis included cataract surgery (n=10; 28.6%), corneal transplant (n=5; 14.3%), and glaucoma surgery (n=3; 8.6%). It was possible to retrieve medical records for some patients (n=22), and we found that most patients were treated with topical fluoroquinolones (n=21) as monotherapy (n=13) or in combination with other antibiotics (n=8). Among 18 patients with recorded final VA outcomes, only a small fraction had a final VA ≥20/200 (n=3), whereas in the others, the final VA scores were “hand motion” (n=6), “light perception” (n=1), or “no light perception” (n=6; including one case with a previously low VA). Among patients with a final VA of “no light perception,” evisceration was performed in three patients, and one patient developed phthisis bulbi.

AC= anterior chamber; HM= hand Smotion; LP= light perception; NLP= no light perception; OD= right eye; OS= left eye; VA= visual acuity.

Antibiotics: AMI= amikacin; CEF= cefalotin; CIP= ciprofloxacin; CLIN= clindamycin; CTX= cefotaxime; DOX= doxycycline; ERY= erythromycin; GAT= gatifloxacin; GEN= gentamicin; MOX= moxifloxacin; OFLOX= ofloxacin; TOB= tobramycin; VAN= vancomycin.

## DISCUSSION

Alpha-hemolytic streptococci are part of a large group of organisms, for which species-level identification is not routinely performed. Generally, clinicians are limited to differentiation between *S. pneumoniae* and the viridans species as a group^(23)^. This creates a gap in our understanding of ocular infections caused by this group of bacteria. Here we sought to determine the species distribution of alpha-hemolytic streptococci causing ocular infections, their associated antibiotic sensitivity profiles, and clinical outcomes. We found nine different species representing the *mitis* and *sanguinis* groups, with *S. oralis, S. pneumoniae, Streptococcus sanguinis,* and *Streptococcus mitis* predominating. Differences were present according to the site of infection. A high number of *S. oralis* isolates were identified in both endophthalmitis and keratitis. *S. sanguinis* was more common in endophthalmitis, *S. pneumoniae* was more common in keratitis, and *S. mitis* was only recovered from keratitis patients.

Microbiological studies have reported that the genus *Streptococcus,* along with the genera *Staphylococcus, Corynebacterium,* and *Propionibacterium*, comprises the commensal bacterial population on the ocular surscies is the upper respiratory tract, which is why it is also speculated that the growth of *S. pneumoniae* in endophthalmitis that develops post-intravitreal injection may be associated with aerosolization of saliva. This may occur more often when the injection is performed in the physician’s office if regular precautions such as mask-wearing or a no-talking rule are not followed^(25)^. It should also be noted that in ophthalmology, the use of preoperative povidone iodine antiseptic in eye preparations is highly recommended, because it is considered effective and economically reasonable and does not induce antibiotic resistance^(26)^.

We found that *S. oralis* was the most prevalent species causing streptococcal endophthalmitis in our population (p=0.0013), followed by *S. sanguinis.* However, most of the endophthalmitis cases included in our study were associated with phacoemulsification surgery, where the route of contamination was expected to differ from that in-office intravitreal injection^(24^,^27)^. When inoculated into the posterior chamber, VGS can cause aggressive and rapidly developing endophthalmitis. In this study, the majority of endophthalmitis infections resulted in very poor final VA, with patients scored as “no light perception” (n=6), “light perception” (n=3), or “hand motion” (n=1). Of the six patients with no light perception, one was subjected to enucleation and another to evisceration, and two resulted in phthisis bulbi. Similar clinical outcomes were described for patients involved in an outbreak of post-intravitreal injection endophthalmitis caused by *S. mitis*/*S. oralis* in southern Florida, where 11 of 12 patients were left with minimal vision with a VA ≤ “hand motion”^(6)^.

In contrast to endophthalmitis, infectious keratitis was predominantly caused by *S. pneumoniae* (p=0.0013) and less frequently by *S. oralis* or *S. mitis. S. pneumoniae* is not only a major cause of conjunctivitis^(22)^ but also a common cause of infectious keratitis. Pneumococcal keratitis is not typically associated with contact lens wear, but predisposing conditions such as ocular trauma or surgery are important risk factors^(28)^. In our population, most patients with pneumococcal keratitis had a history of cataract or glaucoma surgery, corneal transplantation, or trauma. Most of these patients were treated with fluoroquinolones as monotherapy and also had poor visual outcomes.

There was high genetic diversity (SDI=0.982) in the *S. pneumoniae* population studied, with 13 different STs found among 19 isolates (2 isolates from endophthalmitis and 17 from keratitis). All STs belonged to different CCs, except for ST66 and ST73 (CC66). Most of these STs (n=15; 78.9%) tested positive for the *cps*A gene and were involved in the biosynthesis of capsular polysaccharide, which is a key virulence factor; the capsule surrounds the bacterial cell and forms a protective barrier to resist the host immune system^(29)^. Only four isolates (21.1%) tested negative for the *cps*A gene and were therefore predicted to be unencapsulated. These isolates belonged to ST1262 and ST11374 and the conjunctivitis-associated strain ST2315^(22)^. Antibodies against the capsule are the basis of current vaccines that are composed of capsular polysaccharides conjugated to protein (PCV7, PCV10, and PCV13), and epidemiological reports indicate that there is an increasing prevalence of conjunctivitis outbreaks and otitis media infections caused by unencapsulated *S. pneumoniae* strains^(29^,^30)^. In the present study, all but four *S. pneumoniae* isolates tested positive for the *cps*A gene. This may indicate a possible failure of current pneumococcal immunization targeting the common polysaccharide capsular serotypes. Therefore, further studies using a larger population and serotyping information will be necessary to determine if this vaccine fails to prevent ocular pneumococcal infections.

Fluoroquinolones combined or as monotherapy are the most frequently used class of topical antibiotics in the treatment of ocular infections. Although resistance to fluoroquinolones among alpha-hemolytic strepto cocci may arise after exposure, it is still relatively uncommon. In our population, only 7.0% of the VGS isolates were resistant to levofloxacin and 2.3% were resistant to moxifloxacin. All *S. pneumoniae* isolates were susceptible to the fluoroquinolones.

High levels of susceptibility were observed for antibiotics frequently used to treat endophthalmitis, such as cephalosporins (cefotaxime, ceftriaxone, and cefepime) and vancomycin (100%). The cases included in our study were mainly treated with intravitreal injections of vancomycin and ceftazidime, along with topical use of fluoroquinolones and corticoids. Despite the sensitivity of the isolates to the antimicrobials used, most of the patients had poor visual outcomes, demonstrating that virulence factors other than resistance to antibiotics played an important role in the course of eye infections caused by alpha-hemolytic streptococci.

In the present study, the majority of keratitis cases were treated with fluoroquinolones (n=21 out of 22 data recoveries) as monotherapy (n=13) or combined with other antibiotics (n=8). VGS exhibited high susceptibility to cephalosporins, although the *in vitro* susceptibility of VGS recovered from infectious keratitis cases was lower for levofloxacin (93.0%) and moxifloxacin (97.7%).

In conclusion, the distribution of alpha-hemolytic Streptococcus species in ocular infections is not random, suggesting that possible species-specific tissue tropism exists. This finding is consistent with a model in which *S. pneumoniae* is better able to attach to the corneal epithelium and resist local immune defenses at this site, whereas *S. oralis* exhibits a greater capacity to invade the posterior chamber and cause endophthalmitis. Additionally, antibiotic resistance does not seem to be an important contributor to the differential selection of these species in different ocular tissues and is also not likely to play a role in poor clinical evolution and outcomes.
